# Evaluation of Serum and Aqueous Humor Neurofilament Light Chain as Markers of Neurodegeneration in Glaucoma

**DOI:** 10.1167/tvst.14.2.24

**Published:** 2025-02-25

**Authors:** Jonathan B. Lin, Hani El Helwe, Henisk Falah, Bruno L. Hammerschlag, Stephanie A. Schultz, George Baldwin, Yixi Xue, Ryan A. Vasan, Christian Song, Kristine Lo, Austin Meeker, Silas L. Wang, Pia Kivisäkk, David Solá-Del Valle, Milica A. Margeta

**Affiliations:** 1 Department of Ophthalmology, Harvard Medical School and Massachusetts Eye and Ear, Boston, MA, USA; 2 Department of Neurology, Massachusetts General Hospital, Boston, MA, USA

**Keywords:** neurofilament light chain (NfL), glaucoma, neurodegeneration, biomarker

## Abstract

**Purpose:**

The purpose of this study was to evaluate the relationship between serum and aqueous humor (AH) neurofilament light chain (NfL) and to determine whether serum NfL is elevated in patients undergoing ocular surgery who have glaucoma compared with those who do not.

**Methods:**

In this single-center, case-control study, we enrolled patients with various types and stages of glaucoma undergoing planned ophthalmic surgery as part of their routine care and compared them with patients without glaucoma undergoing phacoemulsification for age-related cataract. We recruited 110 patients with glaucoma and 113 patients without glaucoma and collected AH and blood from these participants. Levels of AH and serum NfL were quantified using the Single-Molecule Array (Simoa) NF-light assay (Quanterix). Clinical information was obtained by reviewing the medical records.

**Results:**

In a model controlling for age and body mass index (BMI), AH NfL was significantly elevated in patients with glaucoma compared with controls (*P* < 0.001). In contrast, after controlling for age, BMI, and Mini Mental Status Examination (MMSE) scores, serum NfL was not elevated in patients with glaucoma compared with controls (*P* = 0.81).

**Conclusions:**

Although our findings validate AH NfL as a marker of glaucomatous neurodegeneration, no such evidence was found for serum NfL.

**Translational Relevance:**

NfL levels in AH may be a molecular marker of retinal ganglion cell health in glaucoma; in contrast, serum NfL has limited utility for monitoring glaucomatous neurodegeneration.

## Introduction

Glaucoma is a progressive neurodegenerative disease of retinal ganglion cells (RGCs) and is a leading cause of blindness worldwide, with significant socioeconomic impact.^1^^,^^2^ The exact molecular and cellular mechanisms causing RGC degeneration are areas of active research; however, all current therapies for slowing or preventing RGC degeneration and associated vision loss aim to reduce intraocular pressure (IOP), the only known modifiable risk factor for the disease.^3^ Current strategies for monitoring disease progression rely on surrogate markers of RGC health, such as automated perimetry to detect peripheral vision loss or optical coherence tomography (OCT) to measure the extent of retinal nerve fiber layer (RNFL) thinning.^4^^,^^5^ Nonetheless, these are relatively crude measures that are imperfect for detecting early disease, subtle disease progression, or glaucoma in eyes with other significant ocular comorbidities. Thus, there is a clinical need for more precise methodologies for interrogating RGC health.^6^

An optimal approach for monitoring RGC health would be able to identify subtle cellular dysfunction or early cellular loss that may enable us to prevent substantial RGC damage and associated vision loss. Advanced imaging has emerged as one possible strategy for monitoring RGC health in patients with glaucoma.^7^ For example, one promising technique is the detection of apoptosing retinal cells (DARC), which uses fluorescently labeled annexin V to identify cells destined to undergo apoptosis or programmed cellular death.^8^ This strategy has been shown to be safe in a phase I study, and a subsequent phase II study showed that patients with glaucoma who had more DARC signal subsequently exhibited more RNFL thinning on OCT.^9^ Other strategies have been investigated, such as measuring flavoprotein fluorescence as a proxy for RGC mitochondrial function or using other non-annexin-based peptide probes that detect apoptosis.^10^^–^^12^ These noninvasive methods would be valuable given their ability to detect early cellular changes that may precede irreversible RGC loss.

Another approach is to identify molecular markers within ocular biofluids that reflect underlying RGC stress or dysfunction in glaucoma. Neurofilament light chain (NfL) is a neuronal cytoskeletal protein that has been investigated as a molecular marker of neurodegeneration in the context of numerous neurodegenerative diseases, including Alzheimer's disease, amyotrophic lateral sclerosis, and multiple sclerosis, among others.^13^^–^^15^ Numerous lines of research have shown that NfL is elevated in cerebrospinal fluid, as well as in serum, in patients with ongoing neurodegenerative disease. In two pilot studies, we and others previously reported that aqueous humor (AH) NfL was elevated in patients with glaucoma compared with controls, suggesting that NfL may be a molecular marker of glaucomatous neurodegeneration.^16^^,^^17^ Obtaining AH at the time of planned ocular surgery is safe,^18^ but it is more challenging to obtain AH from patients not undergoing ocular surgery. A blood biomarker for glaucoma would be significantly easier to obtain and, thus, has more potential to be useful in clinical trials and to influence clinical practice. The identification of a novel blood-based molecular marker for glaucoma would revolutionize our ability to care for patients with glaucoma in today's era of precision care.

In this study, we collected AH and serum samples from patients with glaucoma undergoing ocular surgery as part of their routine care to determine whether there is an association between serum and AH NfL. Additionally, we sought to determine whether serum NfL is associated with clinical features of the disease, with the goal of determining whether serum NfL has the potential to serve as a biomarker of glaucomatous neurodegeneration.

## Methods

This single-center, cross-sectional, case-control study was approved by the Mass General Brigham Institutional Review Board and adhered to the tenets of the Declaration of Helsinki. All participants provided written informed consent after explanation of the expected risks and benefits of study participation. Cases included 110 patients with any type of glaucoma treated by 1 of 2 glaucoma specialists (authors D.S. and M.A.M.) who were determined to be candidates for glaucoma surgery as part of their usual clinical care. Controls included 113 patients with age-related cataract and no known diagnosis of glaucoma, glaucoma suspect, or ocular hypertension, who were determined to be candidates for phacoemulsification with intraocular lens implantation and were treated by 1 of 5 comprehensive ophthalmologists (authors R.A.V., C.S., K.L., A.M., and S.L.W.). Eyes were excluded if they had active ocular inflammation, retinal disease, or optic nerve degeneration from non-glaucomatous causes. Patients were excluded if they reported any personal history of neurodegenerative disease or if they reported any unexplained memory problems or neurological symptoms, such as numbness, weakness, or difficulty with ambulation. We allowed both eyes of a patient to be included in the study if they individually met the criteria for inclusion. There were 12 patients (9 with glaucoma and 3 healthy controls) who had both eyes included in the study; all of them had surgery in one eye at a time.

Patients were recruited in the preoperative holding area on the day of their planned ocular surgery. After informed consent was obtained, whole blood was obtained from participants using standard aseptic technique. On the same day that the blood was collected, serum was extracted from whole blood by centrifuging the samples at 2000 rotations per minute for 10 minutes and carefully pipetting the serum away from the pellet. Serum was aliquoted and stored at −80°C until further analysis. The Mini Mental Status Examination (MMSE), an extensively validated, 11-question instrument for assessing global cognition, was performed on all participants prior to the surgery to detect possible subclinical cognitive impairment.^19^ AH was obtained from participants in the operating room at the start of their planned ophthalmic surgery. Briefly, a blunt cannula on a syringe was inserted into the initial peripheral paracentesis and used to remove 25 to 150 µL of AH. The planned ophthalmic surgery was then performed. AH was immediately placed on dry ice and stored at −80°C until further analysis.

To determine the appropriate sample size to identify a difference in serum NfL levels in patients with glaucoma compared with healthy controls, we performed an a priori power analysis using the *pwr* package in R.^20^ Because neurodegeneration from causes unrelated to glaucoma influences serum NfL, we estimated an effect size of f^2^ = 0.11, which represents 45% of the effect size we observed in AH.^17^ Based on this effect size and accounting for the need to control for three covariates (age, body mass index [BMI], and MMSE scores), we calculated the need for 103 subjects per group to achieve 80% power at a 2-tailed alpha level of 0.05. At the completion of the study, we had recruited 113 healthy controls and 110 cases and collected serum and AH from these patients. Some serum and AH samples were used for other pilot studies; as such, serum from 112 controls and 108 cases were available for quantitative analysis, meeting our predetermined study size requirements. Additionally, AH from 79 controls and 99 cases were available for quantitative analysis.

NfL levels in AH and serum were measured using the commercially available single-molecule array (Simoa) NF-light Advantage kit with an automated Simoa HD-X Analyzer (Quanterix, Billerica, MA, USA) at the Mass General Hospital (MGH) Clinical and Translational Research Unit (CTRU) Biomarker Core by an individual masked to the clinical information (author B.L.H.). Simoa is a digital immunoassay that permits ultrasensitive protein detection and has been used to measure NfL and numerous other proteins in various biofluids, including plasma and cerebrospinal fluid.^21^ Samples were randomized across four plates, and three analytical controls were included on each plate. The coefficient of variation (CV) for the analytical controls was 5.4 ± 2.4% (mean ± SD), and no normalization between plates was considered necessary. AH samples were diluted (4× to 48×) in assay diluent depending on available sample volume and assayed in singlicate. As a proof of concept to ascertain whether running AH samples at different dilution factors introduced possible bias, we also ran 3 of the AH samples from patients with glaucoma at 6 different dilution factors and found that there was good dilution linearity across dilution factors from 4× to 48× (Supplementary Fig. S1) with CVs of 5.2%, 6.8%, and 9.8%.

The functional lower limit of quantification (LLOQ) of AH NfL ranged from 1.38 to 16.56 pg/mL, depending on the dilution factor, and the functional upper limit of quantification (ULOQ) ranged from 360 to 17,280 pg/mL. Of the 178 AH samples available for analysis, 19 samples (3 from patients with glaucoma and 16 healthy controls) had NfL levels that were below the LLOQ, and 18 (3 from patients with glaucoma and 15 healthy controls) samples were below the lower limit of detection (LLOD). The 18 samples that were below the limit of detection were omitted from subsequent quantitative analysis, yielding a final number of 160 of AH samples for analysis (96 cases and 64 controls). Supplementary Table S1 shows the distribution of the number of AH samples whose NfL levels were measured below the LLOQ and LLOD across the different dilution factors. Serum samples were diluted to four times and assayed in duplicate. The functional LLOQ of serum NfL was 1.38 pg/mL, and the functional ULOQ was 1440 pg/mL. All 220 serum samples were within this linear range for NfL quantification. The median CV among serum samples run in duplicate was 3.4% (25th percentile = 1.4% and 75th percentile = 5.4%); one serum sample from a patient with glaucoma had a CV > 20% and was omitted from further quantitative analysis, yielding a final number of 219 serum samples (107 cases and 112 controls) for quantitative analysis.

Demographic and clinical information, including age, sex, BMI, self-reported race, laterality, surgery type, glaucoma type, preoperative IOP (taken as the average of the 2 visits prior to their planned ocular surgery), maximum IOP, number of preoperative glaucoma medication classes, Humphrey visual field mean deviation (obtained with 24-2 Swedish Interactive Threshold Algorithm [SITA] Standard test pattern; Humphrey Visual Field HFA 750i, Carl Zeiss Meditec, Dublin, CA, USA), and RNFL thickness measured with the Cirrus HD-OCT 500 (Carl Zeiss Meditec, Dublin, CA, USA), was obtained by retrospective chart review by investigators masked to NfL levels (authors H.E. and H.F.). Glaucoma staging (i.e. mild, moderate, or advanced) was determined based on the International Classification of Disease, 10th revision (ICD-10) criteria.^22^ IOP was measured as a part of routine care with a variety of methods: patients with glaucoma had IOP measured with Goldmann applanation tonometry, whereas the healthy controls had IOP measured with either the Tono-Pen or the iCare tonometer.

We performed statistical analysis and data visualization using R version 4.4.1 and RStudio version 2024.04.02+764. To compare means between two groups, we used the Welch two-sample *t*-test. To determine relationships between categorical variables, we used the Chi square test or the Fisher exact test. To characterize the relationship between continuous variables, we created linear mixed effects models, including age, BMI, and/or MMSE scores as covariates when indicated. For these analyses, AH and serum NfL levels were log_2_-transformed to achieve normality. Linear mixed effects models incorporate both fixed and random effects and therefore allow for the inclusion of two eyes from the same participant in the analysis.^23^ To ensure that imprecise estimations of AH NfL levels for those samples that were below the LLOQ did not bias our results, we performed sensitivity analyses by re-running the analysis comparing AH NfL in patients with glaucoma versus controls while omitting these samples. For all analyses, we considered *P* < 0.05 to be statistically significant. The code used for the analysis herein is available under a GNU General Public License (GPLv3): https://github.com/jonathanblin/nfl-glaucoma.

## Results

Table 1 shows the demographic and clinical characteristics of the participants. There were no significant differences between cases and controls in terms of age, BMI, or laterality. Among cases, there were more participants who self-reported as Hispanic or Black compared with controls. Table 2 shows additional clinical characteristics of the patients with glaucoma. The cases had diverse types of glaucoma and underwent a variety of surgical interventions. Of the patients with glaucoma, 29 (26%) were classified as having a mild stage, 31 (28%) as having a moderate stage, and 50 (45%) as having an advanced stage based on ICD-10 criteria. The patients with glaucoma were on a diverse number of preoperative medication classes prior to surgery.

**Table 1.** Demographic and Clinical Information of Participants

Characteristic	Control	Glaucoma	*P* Value
Total number	113	110	
Age, y, mean (SD^*^)	70.4 (8.7)	68.2 (13.1)	0.1^†^
Sex, *n* (%)			0.9^‡^
Male	45 (40)	45 (41)	
Female	68 (60)	65 (59)	
Body mass index, mean (SD)	28.4 (5.5)	28.5 (7.4)	>0.9^†^
Self-reported race, *n* (%)			<0.001^§^
White	97 (86)	59 (54)	
Black	4 (4)	17 (15)	
Hispanic	6 (5)	26 (24)	
Asian	5 (4)	3 (3)	
Other	1 (1)	5 (5)	
Laterality, *n* (%)			>0.9^‡^
Right	53 (47)	51 (46)	
Left	60 (53)	59 (54)	

* Standard deviation.

† Welch two-sample *t*-test.

‡ Chi square test.

§ Fisher exact test.

**Table 2.** Clinical Characteristics of Patients With Glaucoma

Characteristic	*n* (%)
Surgery type	
Phaco	1 (1)
Phaco/minimally invasive glaucoma surgery (MIGS)	34 (31)
MIGS alone	15 (14)
Incisional	32 (29)
Phaco/incisional	24 (22)
Other	4 (4)
Glaucoma type (%)	
Primary open angle	49 (45)
Normal tension	8 (7)
Mixed mechanism	35 (32)
Pseudoexfoliation	9 (8)
Chronic angle closure	3 (3)
Neovascular	1 (1)
Pigment dispersion	4 (4)
Traumatic	1 (1)
Glaucoma stage (%)^*^	
Mild	29 (26)
Moderate	31 (28)
Advanced	50 (45)
Number of preoperative glaucoma medication classes (%)^†^	
0	2 (2)
1	15 (14)
2	9 (8)
3	19 (17)
4	18 (16)
5	32 (29)
6	14 (13)
7	1 (1)

* Defined by International Classification of Disease, 10th revision (ICD-10) criteria.

† Oral and topical carbonic anhydrase inhibitors were counted as separate classes of glaucoma medications given their different routes of administration.

**Figure 1.**
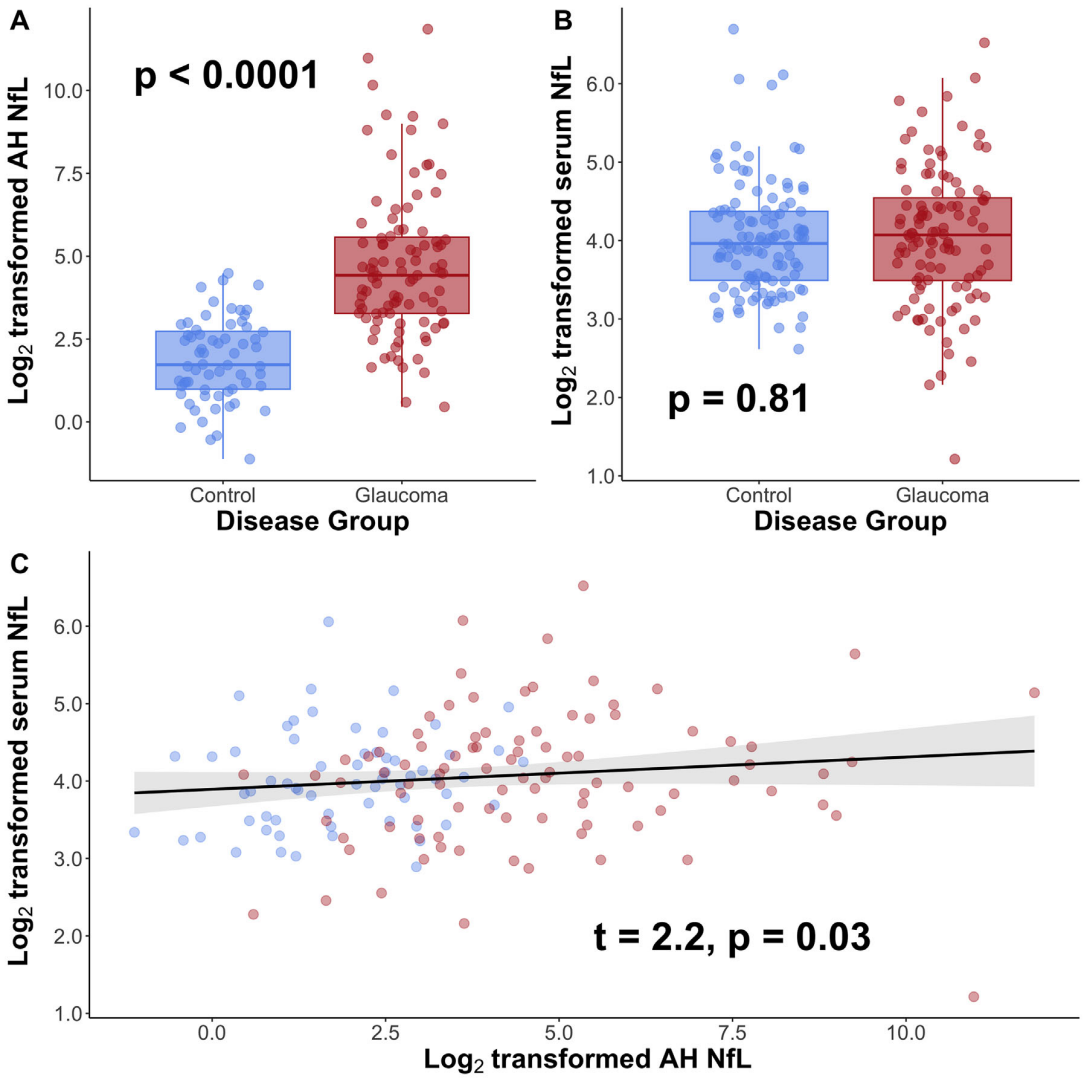
Neurofilament light chain (NfL) is elevated in aqueous humor (AH) but not serum of patients with glaucoma. (**A**) Log_2_-transformed absolute NfL levels (pg/mL) are significantly elevated in AH from patients with glaucoma versus from healthy controls. (**B**) Log_2_-transformed absolute serum NfL levels (pg/mL) are not different in patients with glaucoma versus healthy controls. (**C**) There is a statistically significant association between AH and serum NfL levels among patients with glaucoma and controls. *Individual circles* depict individual participants (**A–C**). The *gray shading* depicts 95% confidence interval bands (**C**).

In a model controlling for age and BMI, log_2_-transformed AH NfL levels were significantly higher in patients with glaucoma compared with controls (*P* < 0.0001; Fig. 1A). To estimate the magnitude of association between AH NfL and glaucoma, we calculated the odds ratio. In a model controlling for the effects of age and BMI, each 2-fold increase in AH NfL was found to be associated with 3.3-fold increased odds of having glaucoma (95% confidence interval [CI] = 2.4 to 5.2, *P* < 0.001). Similar results were obtained when the analysis was performed while omitting the participants for whom AH NfL levels fell above the limit of detection but below the LLOQ (data not shown). In a model controlling for age, BMI, and MMSE score, log_2_-transformed serum NfL levels were not different when comparing patients with glaucoma with healthy controls (*P* = 0.81; Fig. 1B). We also calculated the association between serum and AH NfL. In a model controlling for the effects of age, BMI, and MMSE scores, there was a significant, albeit weak, association between log_2_-transformed serum NfL and log_2_-transformed AH NfL (t = 2.2, *P* = 0.03; Fig. 1C): each 2-fold increase in AH NfL was associated with a 4% increase in serum NfL.

**Figure 2.**
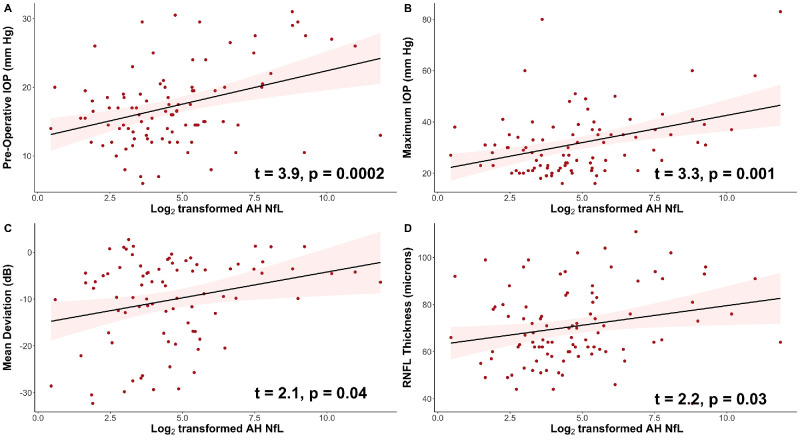
In patients with glaucoma, there are moderate positive associations between levels of aqueous humor (AH) neurofilament light chain (NfL) and (**A**) preoperative intraocular pressure (IOP), (**B**) maximum IOP, (**C**) mean deviation on Humphrey visual field testing, and (**D**) retinal nerve fiber layer (RNFL) thickness, as measured by optical coherence tomography (OCT). *Individual circles* depict individual participants; the *red shading* depicts 95% confidence interval bands (**A–D**).

**Figure 3.**
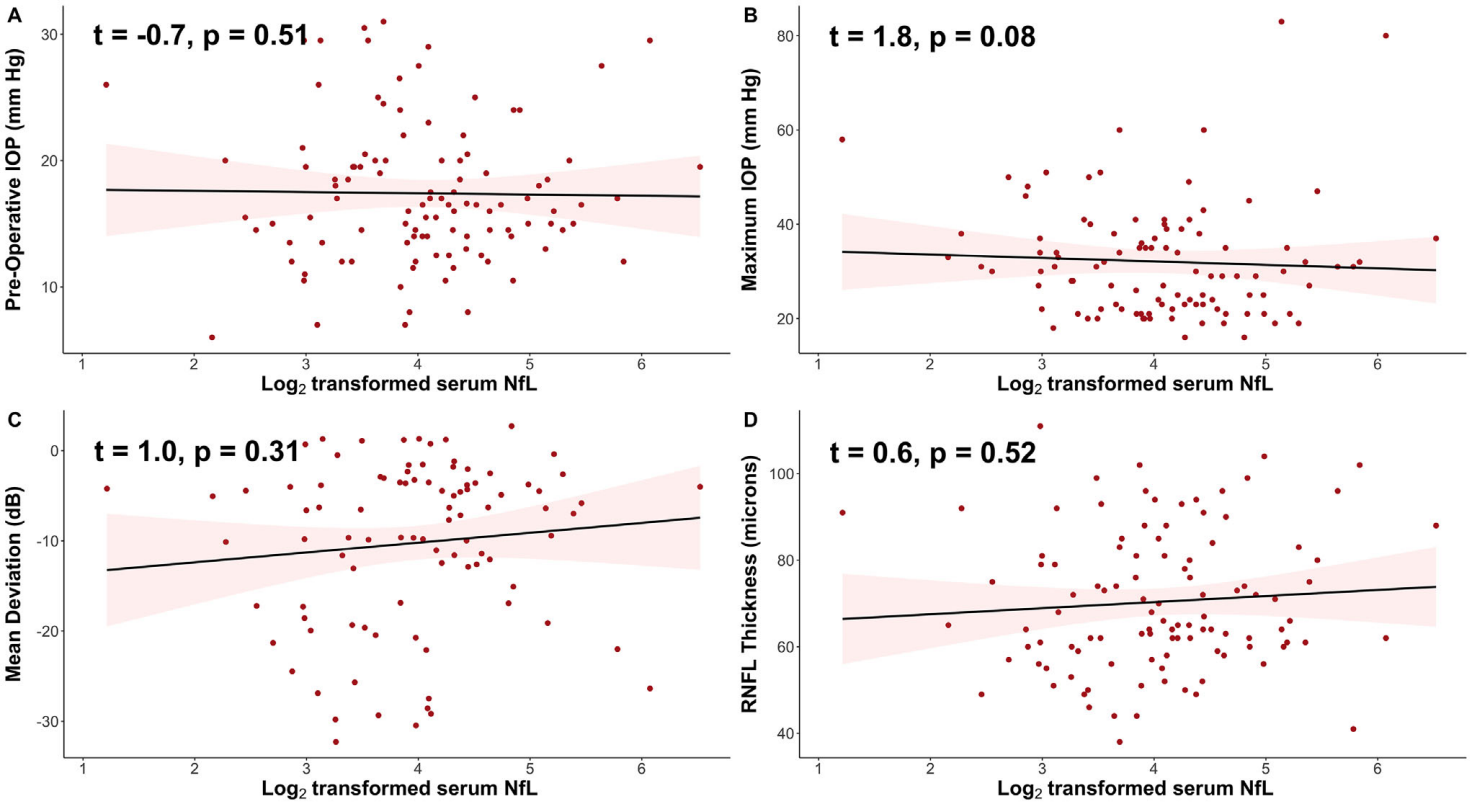
In patients with glaucoma, there are no significant associations between levels of serum neurofilament light chain (NfL) and (**A**) preoperative intraocular pressure (IOP), (**B**) maximum IOP, (**C**) mean deviation on Humphrey visual field testing, and (**D**) retinal nerve fiber layer (RNFL) thickness, as measured by optical coherence tomography (OCT). *Individual circles* depict individual participants; the *red shading* depicts 95% confidence interval bands (**A–D**).

We also sought to determine whether, among patients with glaucoma, elevated AH NfL was associated with clinical characteristics of glaucoma. After controlling for age and BMI, log_2_-transformed AH NfL levels were positively associated with preoperative IOP (t = 3.9, *P* = 0.0002; Fig. 2A) and maximum IOP (t = 3.3, *P* = 0.001; Fig. 2B). Somewhat paradoxically, log_2_-transformed AH NfL levels were also positively associated with mean deviation (MD) on automated perimetry (t = 2.1, *P* = 0.04; Fig. 2C) and RNFL thickness as measured with OCT (t = 2.2, *P* = 0.03; Fig. 2D). Additionally, we sought to determine whether serum NfL was associated with clinical characteristics of glaucoma. After controlling for age, BMI, and MMSE scores, log_2_-transformed serum NfL levels were not significantly associated with preoperative IOP, maximum IOP, MD on automated perimetry, or RNFL thickness (Figs. 3A–D).

Finally, we sought to determine whether, among patients with glaucoma, the number of classes of preoperative glaucoma medications was associated with AH or serum NfL levels. After controlling for covariates, there was no association between the number of preoperative classes of glaucoma medications and either log_2_-transformed AH NfL levels (t = 1.0, *P* = 0.32; Supplementary Fig. S2A) or log_2_-transformed serum NfL levels (t = 1.1, *P* = 0.26; Supplementary Fig. S2B).

## Discussion

In this paper, we found that AH NfL is elevated in patients with glaucoma compared with healthy controls. These findings are in agreement with our prior, smaller case-control study of 39 patients with glaucoma compared with 10 healthy controls taken from a North American patient population,^17^ as well as a similar study conducted in a European (Austrian) patient population.^16^ NfL is a neuronal cytoskeletal protein that is expressed in RGC axons; taken together, our data support the idea that glaucomatous neurodegeneration leads to NfL release from dying RGCs and accumulation of NfL in surrounding ocular biofluids.^24^^,^^25^ Therefore, AH NfL may provide us with the ability to objectively ascertain RGC health at the molecular level in patients with glaucoma.

The main difference between the present study and our prior study^17^ is that we also quantified serum NfL in our participants. In contrast to the significant difference seen in AH NfL, we did not find a significant difference in serum NfL in patients with glaucoma compared with healthy controls after controlling for the effects of age, BMI, and MMSE scores to account for the possibility of undiagnosed mild cognitive impairment. Our study was powered to detect a difference in serum NfL in patients with glaucoma that was 45% of that which we observed in AH NfL. One interesting finding in our study is that despite the absence of a significant elevation in serum NfL in patients with glaucoma, there was a statistically significant association between serum and AH NfL, suggesting that there is some ocular contribution to the systemic pool of serum NfL. However, this contribution appears to be relatively small; in our study, a twofold increase in AH NfL roughly corresponded to a 4% increase in serum NfL. These findings are consistent with prior proteomic studies showing that in patients with retinal diseases, there is a strong association between the proteins detected in aqueous and vitreous humor but much less association with their levels in the serum.^26^ Whereas serum NfL may have utility as a molecular marker for neurodegeneration of the brain and spinal cord for patients with Alzheimer's disease, traumatic brain injury, multiple sclerosis, and Parkinson's disease,^13^^,^^27^^–^^30^ its clinical utility in glaucoma, and ocular disease more broadly, may be limited.

In our study, we also found statistically significant associations between AH NfL and preoperative IOP and maximum IOP, similar to our prior smaller study.^17^ These are clinical factors that tend to be present in poorly controlled disease, and, once again, these findings suggest that AH NfL may be more elevated when there is ongoing RGC damage due to inadequate disease control and unacceptably high IOP. Similar positive associations between AH NfL and IOP were reported in a proteomic study of 36 patients with glaucoma undergoing planned glaucoma filtration surgery versus 35 patients without glaucoma undergoing cataract surgery; in fact, in their unbiased proteomic analysis, AH NfL had the highest correlation with IOP.^31^ We also observed a statistically significant positive association between AH NfL and MD on automated perimetry and RNFL thickness on OCT. These findings initially seem somewhat paradoxical because they suggest that there is higher AH NfL in patients with less glaucomatous damage. However, we speculate that this association is likely because patients with advanced glaucoma may exhibit the floor effect, that is, there are fewer RGCs remaining and therefore less NfL available to be released.

Finally, in contrast with our prior smaller study,^17^ we did not observe a significant association between AH NfL and the number of preoperative classes of glaucoma medications. We previously hypothesized that the positive association observed between these factors may be related to the fact that patients on more preoperative medications may have poorly controlled disease and, thus, have more ongoing RGC degeneration. However, our cross-sectional study design is not optimal for testing this hypothesis, and as such, future longitudinal studies are necessary to directly assess whether there is a link between AH NfL and quantifiable RGC degeneration. Because there is no perfect gold standard for quantifying RGC death, these studies may require using a surrogate measure of RGC health, such as measuring progression over time on automated perimetry, measuring RNFL thinning with OCT, or quantifying future RGC death with advanced imaging techniques, such as DARC.^8^^,^^9^

Strengths of our study include its robust study size. To our knowledge, ours is the largest case-control study investigating NfL as a possible molecular marker of glaucomatous neurodegeneration, and the sample size was determined a priori to detect a clinically significant difference between the groups. In addition, to control for possible influence of subclinical cognitive impairment, we performed the MMSE and controlled for this within our statistical analysis. Our study also had several limitations. Due to its cross-sectional design, we were not able to establish a direct link between ongoing glaucomatous neurodegeneration and elevated AH NfL. Moreover, although we performed an a priori power analysis, the effect size observed in the serum appears to be much smaller than originally anticipated. Therefore, we cannot rule out the possibility that there may indeed be a significant difference in serum NfL in patients with glaucoma versus controls, but such a small magnitude of effect would likely be too small to be clinically useful. Finally, there was a significant difference in the distribution of self-reported race when comparing cases with controls. Although this difference in racial distribution reflects the underlying racial diversity of patients with glaucoma, it remains possible that this difference may have confounded our results. Prior work investigating plasma NfL within racially diverse cohorts suggest that the relationship between systemic NfL and medical comorbidities may be affected by race.^32^

Taken together, this study serves as validation that AH NfL shows promise as a marker of glaucomatous neurodegeneration, whereas serum NfL likely has limited clinical utility given the relatively small contribution of the eye to the systemic circulation. Further longitudinal studies investigating the association of AH NfL and glaucomatous progression are needed to determine the potential utility of AH NfL as a glaucoma biomarker in clinical trials and clinical practice.

## Supplementary Material

Supplement 1
