# Supplementary material for: Evaluation of Serum and Aqueous Humor Neurofilament Light Chain as Markers of Neurodegeneration in Glaucoma
Source: Transl Vis Sci Technol. 2025 Feb 25;14(2):24. doi: 10.1167/tvst.14.2.24 (PMC11875033; doi:10.1167/tvst.14.2.24)
Supplement: Supplement 1 [file tvst-14-2-24_s001.pdf]

SUPPLEMENTARY MATERIALS

**Supplementary Figure S1**. Dilution linearity of aqueous humor neurofilament light chain (NfL) in three glaucoma patients. The shaded area represents 10% relative error from the global mean across the six dilution factors.

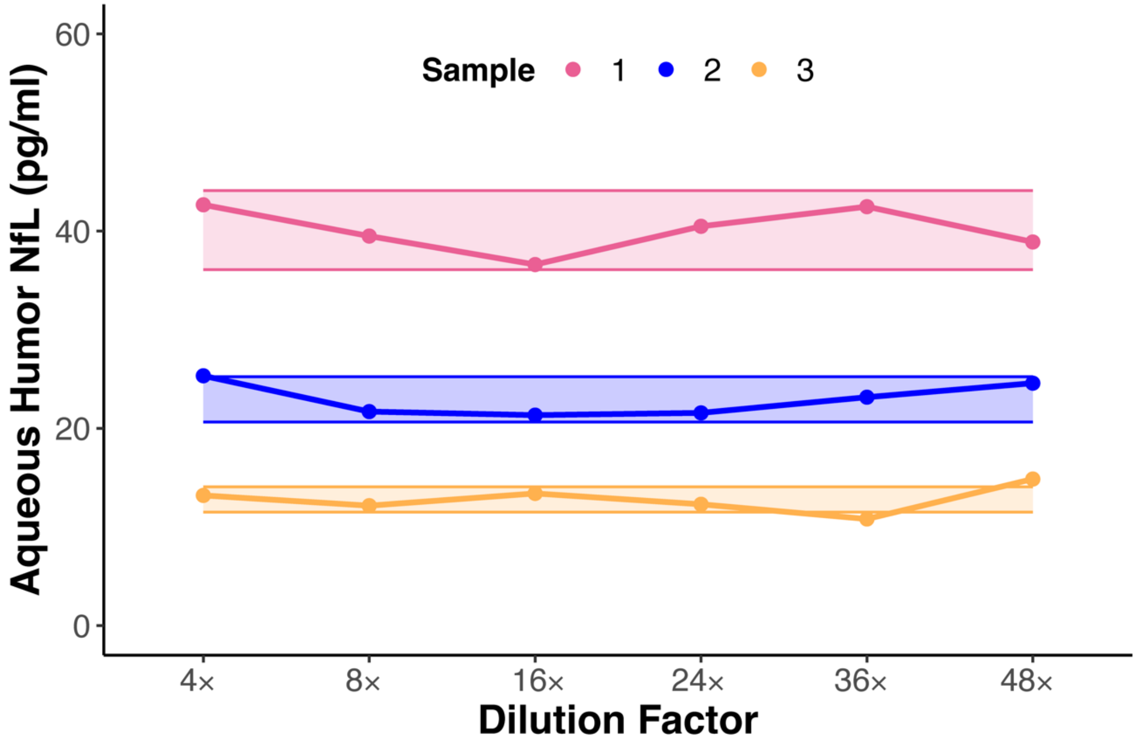

**Supplementary Figure S2**. In glaucoma patients, there were no significant associations between number of classes of pre-operative glaucoma medications and either (A) aqueous humor (AH) neurofilament light chain (NfL) or (B) serum NfL. Individual circles depict individual participants.

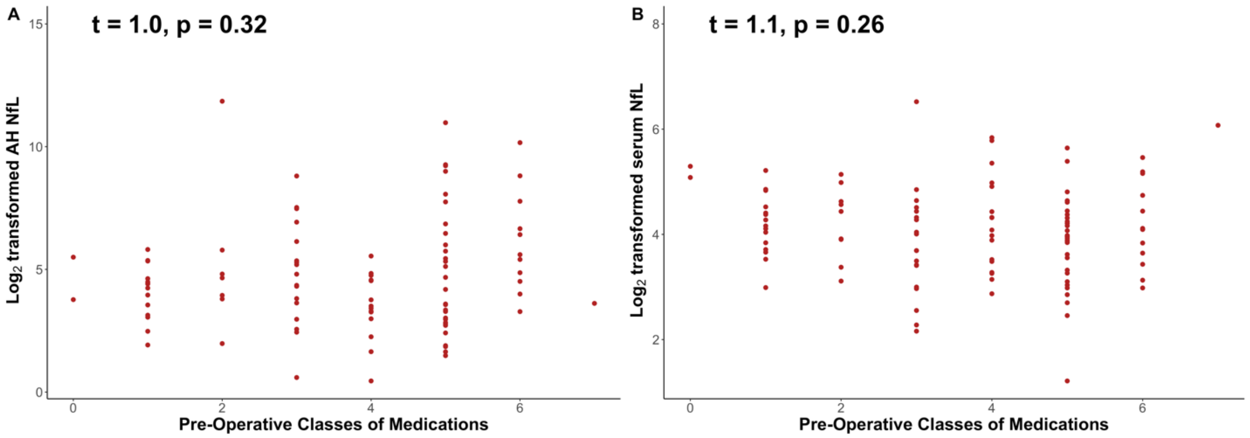

**Supplementary Table S1**. Summary of Quality Control (QC) Parameters for Aqueous Humor Samples.

| **Dilution Factor** | **Control** | | | **Glaucoma** | | |
|---|---|---|---|---|---|---|
| | Total | Below Lower Limit of Detection (LLOD) | Below Lower Limit of Quantification (LLOQ) | Total | Below Lower Limit of Detection (LLOD) | Below Lower Limit of Quantification (LLOQ) |
| **4×** | 47 | 0 (0%) | 4 (9%) | 87 | 1 (1%) | 3 (3%) |
| **8×** | 11 | 0 (0%) | 7 (64%) | 6 | 0 | 0 (0%) |
| **16×** | 5 | 2 (40% | 3 (60%) | 3 | 1 (1%) | 0 (0%) |
| **24×** | 6 | 4 (67%) | 2 (33%) | 2 | 1 (1%) | 0 (0%) |
| **36×** | 6 | 5 (83%) | 0 (0%) | 1 | 0 (1%) | 0 (0%) |
| **48×** | 4 | 4 (100%) | 0 (0%) | 0 | 0 (0%) | 0 (0%) |
| **All** | 79 | 15 (19%) | 16 (20%) | 99 | 3 (3%) | 3 (3%) |
